# Cognitive behavioral therapy versus general health education for family caregivers of individuals with heart failure: a pilot randomized controlled trial

**DOI:** 10.1186/s12877-022-02996-7

**Published:** 2022-04-05

**Authors:** Boyoung Hwang, Douglas A. Granger, Mary-Lynn Brecht, Lynn V. Doering

**Affiliations:** 1 College of Nursing & Research Institute of Nursing Science, Seoul National University, Seoul, South Korea; 2 Institute for Interdisciplinary Salivary Bioscience Research, University of California, Irvine, CA USA; 3 School of Nursing, University of California, Los Angeles, CA USA

**Keywords:** Cognitive behavioral therapy, Heart failure, Family caregiver, Psychological distress, Cortisol

## Abstract

**Background:**

While support from family caregivers is essential in the care of patients with heart failure (HF), caregiving places a considerable burden on family caregivers. We examined the preliminary effects of cognitive behavioral therapy (CBT) for caregivers of individuals with HF.

**Methods:**

In this pilot randomized controlled trial, patients with HF and their primary family caregivers (30 dyads) were randomized into CBT (*n* = 15) or general health education (GHE, *n* = 15) groups. Caregivers received 8 weekly individual sessions of either CBT (intervention) or GHE (attention control condition). Caregivers completed questionnaires at baseline, post-intervention, and 6 months. Saliva samples collected from caregivers at baseline and post-intervention were analyzed for salivary cortisol. The cortisol awakening response (CAR) and area under the curve (AUC) were calculated using log-transformed cortisol values. We analyzed data from 26 (14 receiving CBT and 12 receiving GHE) caregivers who received at least one session of CBT or GHE (modified intention-to treat) using linear mixed models. Each model included time, study group, and time-by-study group interaction as fixed effects.

**Results:**

Patients were older (66.94 ± 14.01 years) than caregivers (55.09 ± 15.24 years), and 54% of patients and 54% of caregivers were female. Most caregivers (58%) were spouses. A total of 14 (93%) CBT and 12 (80%) GHE participants received at least 1 session (*p* = .60), and 11 (73%) CBT and 11 (73%) GHE participants completed all 8 sessions (*p* = 1.00). There were no significant between-group differences in change for salivary cortisol or psychological outcomes. However, the CBT group had significant within-group improvements in perceived stress (*p* = .011), stress symptoms (*p* = .017), depression (*p* = .002), and anxiety (*p* = .006) from baseline to post-intervention, while the control group had no significant within-group change in the outcomes except for anxiety (*p* = .03). The significant improvements observed in the CBT group lasted for 6 months. No adverse effects were observed.

**Conclusions:**

In this pilot trial, although between-group differences in change were not significant, CBT resulted in significant improvements in some psychological outcomes with no improvement in the control group. Our findings suggest the potential of the intervention to alleviate psychological distress in HF caregivers. Further examination in larger randomized trials is warranted.

**Trial registration:**

ClinicalTrials.gov Identifier: NCT01937936 (Registered on 10/09/2013).

## Background

With a continued increase in the prevalence of heart failure (HF), 6 million Americans currently suffer from the disease [1]. Because patients with HF often have difficulty performing daily activities due to their symptoms, such as fatigue, dyspnea, and edema, the increase in HF prevalence inevitably leads to an increase in the number of individuals providing care for their family members with HF. In addition, the need for family caregivers is increasing as the emphasis on self-management and adherence to complex therapeutic regimens in the care of patients with HF increases [2]. While support from family caregivers is crucial for better outcomes for HF patients, caring for a chronically ill family member can be challenging and stressful. HF caregivers have been reported to experience stress, depression, anxiety, and social isolation due to their care responsibilities, perceive their physical and mental health to be compromised, and suffer from poor quality of life [3].

Given the accumulating evidence that chronic stress related to caregiving negatively affects caregivers’ health, many efforts have been made to develop psychosocial interventions to reduce the detrimental effects of stress on caregiver health [4]. Intervention strategies include educational interventions, cognitive behavioral therapy (CBT), mindfulness-based interventions, support groups, and respite care. Although effect sizes varied by study factors, such as caregiver characteristics, intervention settings, and outcome measures used, findings of systemic reviews and meta-analyses consistently indicate that educational interventions and CBT were effective on multiple outcomes (i.e., stress, depression, and subjective well-being) whereas others had domain-specific effects [4, 5]. Yet, most caregiver intervention studies focused on caregivers of patients with dementia, Alzheimer’s disease, and cancer; there have only been a few intervention studies aimed at alleviating stress in HF caregivers [2].

Caregiving can cause chronic stress. Psychological stress responses begin with the perception of an event as stressful. The cognitive evaluation then initiates activation of the hypothalamic-pituitary-adrenal (HPA) axis, which regulates secretion of glucocorticoids [6]. Elevated cortisol levels exert negative feedback effects on the hypothalamus and pituitary, inhibiting the production of corticotrophin releasing hormone and adrenocorticotropic hormone [6]. While the release of cortisol in response to an acute stressor is considered as an adaptive mechanism to enhance survival, chronic elevations of cortisol can have damaging effects on the body over time [7]. Therefore, assessing stress in caregivers using both physiological (i.e., cortisol) and psychological measures (i.e., self-reports) can contribute to a comprehensive understanding of stress related to caregiving.

Salivary cortisol has been widely used in research as a biomarker for stress because the collection of saliva is convenient, painless, and non-invasive [6]. In addition, measuring the activity of the HPA axis via salivary cortisol has been validated extensively [8]. Cortisol follows a strong circadian rhythm, with cortisol levels peaking 30 to 45 min after awakening [i.e., cortisol awakening response (CAR)] and being lowest around midnight [6, 9]. Therefore, in order to capture the diurnal pattern of cortisol secretion accurately, collecting 3 to 4 samples per day for at least 2 consecutive days is recommended [9]. In previous studies, altered diurnal patterns of cortisol secretion and increased cortisol levels were found in caregivers of patients with dementia [10]. And, an increased CAR was reported to be associated with chronic life stress [11, 12]. However, salivary cortisol was used to evaluate the effects of caregiver interventions in only a limited number of studies and diurnal variations in cortisol levels were rarely measured in those studies [10, 13, 14].

To fill these gaps in the existing literature, we conducted a pilot randomized controlled trial to evaluate the potential of an intervention designed to reduce stress using CBT among caregivers of patients with HF. The specific aims of this study were to examine the effects of an 8-week CBT intervention compared to an attention control condition on physiological (i.e., salivary cortisol) and psychological outcomes in HF caregivers at post-intervention and 6-month follow-up and to describe effect sizes. We hypothesized that the CBT group would have greater decreases in cortisol levels and the CAR and greater improvements in psychological outcomes (i.e., caregiver burden, perceived stress, stress-related symptoms, depression, and anxiety) compared to the control group. The psychological outcomes were selected because they are the most common targets of CBT-based interventions for caregivers [5].

## Methods

### Study design and participants

This study was a pilot randomized controlled trial, and the study reporting followed the Consolidated Standards of Reporting Trials (CONSORT) statement [15]. Upon approval from the Institutional Review Board, HF patients and their caregivers were recruited from inpatient units and outpatient clinics at two university-affiliated hospitals located in Los Angeles, California. Patients who were interested in and eligible to participate in this study were asked to identify the person who was primarily involved in their care at home (i.e., primary caregiver). If the caregiver was present, information about this study was provided to the caregiver by study personnel. If not, a call was scheduled to provide information to the caregiver. When both patient and caregiver met the inclusion/exclusion criteria and agreed to participate, written informed consent was obtained from each of them. Study participation was voluntary for all patients and caregivers in our study.

The inclusion criteria for patients were: 1) 21 years or older, 2) primary or secondary diagnosis of HF (New York Heart Association [NYHA] class II–IV), 3) having a family member providing care for them at home, and 4) able to communicate in English. Patients were excluded, if they were: 1) on the transplant list or 2) diagnosed with a terminal illness (e.g., terminal cancer). The inclusion criteria for caregivers were: 1) 21 years or older, 2) primarily responsible for the care of the patient for at least 6 months, 3) living in the greater Los Angeles area, and 4) able to communicate in English. Caregiver burden or psychological distress (i.e., stress, depression, or anxiety) was not required for participant eligibility. Caregivers were excluded, if they were: 1) a paid caregiver, 2) diagnosed with a terminal illness (e.g., terminal cancer) or a serious medical condition that required ongoing medical care (e.g., cancer requiring ongoing chemotherapy or radiation therapy), 3) having a history of major psychiatric illness (i.e., schizophrenia, bipolar disorder, and/or any personality disorder), 4) diagnosed with Cushing’s or Addison’s disease, or 5) taking medication that could influence cortisol levels (e.g., steroid based anti-inflammatory drugs).

### Sample size

We planned to recruit 50 patient-caregiver dyads (25 dyads in each group). Sample size determination was guided by appropriateness for pilot study objectives rather than for formal testing of efficacy hypotheses [16, 17]. The sample size was expected to allow detection of an adjusted effect size of 1.23 in any outcome with power of .80 and 2-tailed alpha of .05, comparing the patterns of change over time using random effects regression for repeated measures and assuming a moderate correlation of .5 and up to 10% attrition [18].

### Randomization

Dyads of HF patients and their caregivers were randomized in a 1:1 ratio into two study groups: CBT group and general health education (GHE) group. A statistician who was not involved in the recruitment process prepared the random allocation sequence using permuted block randomization with a block size of 6–8. Allocations were concealed in sequentially numbered envelopes that were opened only after informed consent was obtained from each dyad. Healthcare providers were blinded to the study group assignment.

### Cognitive behavioral therapy

Caregivers in both groups received usual care. Usual care was defined as routine discharge planning and education for patients and families and home health care if the patient was referred by his/her healthcare provider. In addition, caregivers in the CBT group participated in 8 weekly individual sessions of CBT, which lasted approximately 1 h each. The intervention took place in the caregiver’s home. All sessions were conducted by the first author (BH) who was trained in CBT (Beck Institute, Philadelphia, PA, USA).

The therapy protocol was based on standard CBT manuals [19, 20]. Therapy goals were established with each caregiver in early sessions. Early sessions focused on explanations of the cognitive model and strategies and emphasized the rationale for identifying and changing negative automatic thoughts and for behavioral assignments and homework [19, 20]. At the end of each session, a copy of a summary of the session and homework was given to the caregiver. Homework was reviewed at each session with an emphasis on problems and accomplishments over the past week. Table 1 presents representative content for the sessions, which was used as a guide. In the first 4 sessions and as needed thereafter, caregivers were provided with written education material about HF care (e.g., signs and symptoms of HF, treatment of HF, and HF self-management strategies, including diet and fluid restrictions) published by the Heart Failure Society of America to address the specific needs of caregiving [21]. To ensure intervention fidelity, sessions were audio recorded and reviewed periodically by a senior author (LVD). Written informed consent was obtained from caregivers for the optional audio recording of sessions. After each session, the therapist (BH) completed the treatment process log to document the implementation of the therapy protocol and track the caregiver’s progress.

**Table 1:** Weekly agenda of intervention sessions

**Week 1 Focus: Establish therapeutic relationship** • Establish rapport • Discuss caregiver expectations about therapy and socialize caregiver to therapy • Describe cognitive model and explain strategies, with emphasis on rationale for behavioral assignments and homework • Explain the nature and consequences of stress • Provide summary and elicit feedback from caregiver	
**Weeks 2–3 Focus: Behavioral activation, active problem-solving, and automatic thoughts** • Brief update and bridge from previous session • Prepare agenda • Review homework assignments • Discuss problems and accomplishments since previous session • Instruct caregiver in identifying negative automatic thoughts • Elicit automatic thoughts, specifically in relation to homework assignments • Prepare homework assignments and elicit feedback regarding today’s session	
**Weeks 4–6 Focus: Automatic thoughts and self-therapy** • Continue to identify negative automatic thoughts • Continue to work on rational responses to automatic thoughts • Give additional homework assignments and elicit feedback regarding today’s session	
**Weeks 7–8 Focus: Automatic thoughts, self-therapy, and relapse prevention** • Prepare caregiver for termination of therapy • Emphasize continuation of practicing strategies after termination • Delineate anticipated problems and rehearse coping strategies • Introduce and practice self-therapy techniques • Provide closure and elicit feedback from caregiver	

### General health education (attention control condition)

In addition to the usual care, the GHE group received 8 weekly sessions of GHE on selected topics (e.g., nutrition, healthy food choices, healthy weight, physical activity, sleep, bone health, and oral health) provided by a research assistant who had no experience in CBT. Each educational session lasted approximately 1 h and took place in participants’ home. Discussions during the education sessions were limited to the selected topics.

### Procedures

Baseline data were collected from both patients and caregivers. Caregivers received 8 weekly sessions of either CBT (CBT group) or GHE (GHE group) and repeated the questionnaires at post-intervention (2 months) and 6 months after baseline. Saliva samples were collected from caregivers at baseline and 2 months. Written educational materials and a list of resources were provided to caregivers in the GHE group after completing the 6-month follow-up assessment. Upon completion of each set of questionnaires, participants in both groups were compensated for their time and effort with a $30 gift card.

At baseline, patients completed self-administered questionnaires on sociodemographics. Clinical data of patients were collected from medical records. The severity of symptoms in patients was assessed using the NYHA class. It is based on the extent to which symptoms limit the patient’s level of physical activity [22]. Data on patients’ comorbidities were abstracted from medical records using the Charlson Comorbidity Index [23], which generates a weighted index based on 17 indicators of coexisting conditions. The index takes into account the number and severity of comorbid diseases [23].

### Primary outcomes (salivary cortisol)

Saliva samples were collected from each caregiver at baseline and post-intervention (2 months). Using the Salivette device with a 1 × 4 cm (ployethlyene/styrene) foam swab, saliva samples were collected: 1) on waking; 2) 30 min later; and 3) at bedtime for two consecutive weekdays. Caregivers were instructed not to eat, drink, or brush their teeth for 30 min nor smoke for 60 min prior to each sample collection. An instruction sheet for sample collection was provided. To enhance adherence to sample collection procedures, the study team also sent reminders to caregivers via calls or text messages. Caregivers completed self-report questionnaires on each collection day.

Saliva samples were immediately frozen at caregivers’ homes and collected by study personnel. Universal precautions were followed by all personnel handling saliva samples. Saliva samples were kept frozen until shipped in batches to the lab on dry-ice via overnight delivery. Assays were conducted at the Johns Hopkins University Center for Interdisciplinary Salivary Bioscience Research (Baltimore, MD). On the day of assay, samples were centrifuged at 3000 rpm for 15 min to remove mucins. Saliva was assayed for cortisol using commercially available immunoassays without modifying the manufacturer’s recommended protocol [24]. All samples were assayed in duplicates. The sample test volume was 25 μl, and the range of sensitivity was from .007 to 3.0μg/dL. Average intra- and inter assay coefficients of variation were less than 5 and 10%, respectively.

For cortisol values, outliers, defined as values that are more than 4 standard deviations away from the mean, need to be screened and excluded [25]. Cortisol values for 2 samples were deleted from the data set before analysis. Summary measures of cortisol that were used in the analysis included: 1) the increase (slope) between awakening and the peak after 30 min, the CAR [9, 26]; and 2) the area under the curve with respect to ground (AUC_G_) calculated by trapezoidal estimation, which represents the total output of cortisol across the day [27]. Average values from the duplicate assays were used in the analysis. Prior to calculation, cortisol values were log transformed because of the skewed distribution. The CAR was computed as the difference in log-transformed cortisol values from waking to 30-min post-awakening for each day. The CAR and AUC were calculated separately for each day and averaged across days for analyses [26, 27].

### Secondary outcomes (psychological outcomes)

Secondary outcomes (psychological outcomes) included caregiver burden, perceived stress, stress-related symptoms, depression, and anxiety and were measured using self-report questionnaires. The level of caregiver burden was measured with the Zarit Burden Interview (ZBI) [28], which consists of 12 items. Scores can range from 0 to 48 with higher scores indicating greater caregiver burden [28]. The ZBI is one of the most frequently used measures of caregiver burden and has been validated in various chronic conditions [29]. It showed acceptable internal consistency in our sample (Cronbach’s alpha = .90). The 10-item Perceived Stress Scale (PSS-10) [30] is the most widely used and valid instrument for measuring the perception of stress, and it measures the degree to which individuals perceive their lives to be stressful and uncontrollable [30, 31]. Cronbach’s alpha in our sample was .93. The Calgary Symptoms of Stress Inventory (cSOSI) [32] measures physical, psychological, and behavioral responses to stressful situations. Caregivers were instructed to rate the frequency with which they experience various stress-related symptoms during the past week. The questionnaire contains 56 items, each of which is rated on a 5-point Likert scale ranging from 0 (never) to 4 (very frequently) [32]. The psychometric properties of the cSOSI have been established [32]. Depressive symptoms of caregivers were measured with the 9-item Patient Health Questionnaire (PHQ-9) [33]. The PHQ-9 is a brief tool for depression screening. Possible scores range from 0 to 27, and a score greater than or equal to 10 suggests clinical depression [33]. Its reliability and validity have been thoroughly examined [34]. In our sample, the internal consistency of the PHQ-9 was good (Cronbach’s alpha = .91). The Generalized Anxiety Disorder 7-item (GAD-7) [35] was used to measure anxiety symptoms of caregivers, and the possible scores range from 0 to 21 [35]. The reliability and validity of the GAD-7 have been well established in the primary care setting [34, 35]. In our sample, Cronbach’s alpha of the GAD-7 was .93.

### Statistical analysis

Data were analyzed using IBM-SPSS 23 (IBM Corporation, Armonk, NY) and SAS 9.4 (SAS Institute, Gary, NC). Analyses were conducted following the modified intention-to-treat principle, and therefore, included data from all dyads whose caregiver received at least one session of either CBT or GHE. Characteristics of HF patients and caregivers and other study variables were summarized using descriptive statistics including means, standard deviations, frequencies, and ranges. Baseline characteristics of patients and caregivers in the 2 study groups were compared using independent t-tests, Mann–Whitney U tests, chi-square tests, or Fisher’s exact tests, as appropriate.

To evaluate preliminary effects of the intervention on outcomes of HF caregivers, we conducted linear mixed models with restricted maximum likelihood estimation (SAS Proc MIXED) and an autoregressive covariance structure. In each model, time, study group, and time-by-study group interaction were included as fixed effects. To control for the differences in baseline caregiver characteristics between the two groups, we also ran each model with the baseline variables that were significant or marginally significant (*p* < .01) and compared the results. All assumptions for each statistical test were checked, and the statistical significance level was set at .05 for all analyses. Additionally, an effect size (Cohen’s *d*) for the difference between groups in the change scores was calculated for each outcome [36]. According to Cohen [36], effect size values of 0.2, 0.5, and 0.8 are interpreted as small, medium, and large, respectively.

## Results

### Recruitment and baseline characteristics

**Fig. 1:**
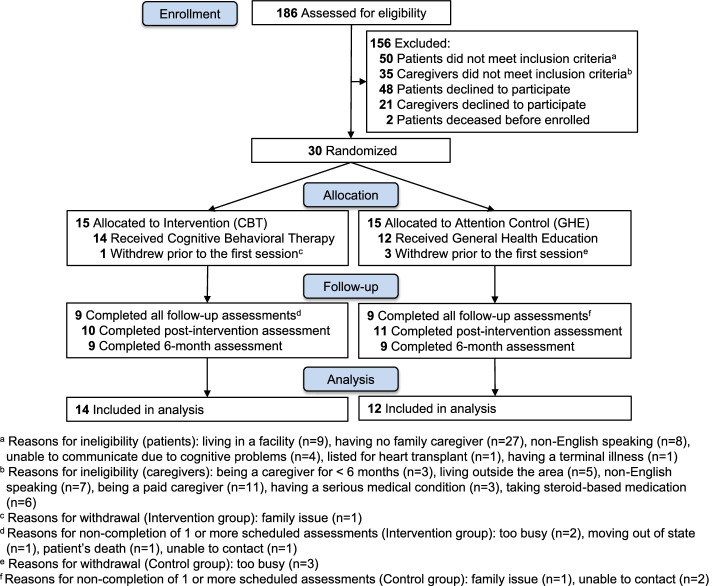
Flow of enrollment, randomization, and follow-up

Recruitment was conducted from August 2013 through September 2014, and the last 6-month follow-up was completed in February 2015. We approached 186 dyads of patients with HF and their primary caregivers (Fig. 1). Among those, 85 dyads were ineligible for the study.

Out of the 101 eligible dyads, 69 were excluded because either the patient (*n* = 48) or caregiver (*n* = 21) declined to participate in the study. Additionally, two dyads were excluded because patients deceased before study enrollment. The remaining 30 dyads (29.7% of the eligible dyads, 60% of the target sample size) were enrolled and randomized. Among the 30 randomized caregivers, 12 (80%) in the GHE and 14 (93%) in the CBT groups received at least 1 session (*p* = .60). Data from the 26 dyads who received at least 1 session of either health education or CBT were included in the analysis.

Demographic characteristics of patients and caregivers in the two groups are presented in Table 2. On average, patients were older (66.94 years old [SD 14.01]) than caregivers (55.09 years old [SD 15.24]), and 14 (54%) patients and 14 (54%) caregivers were female. Compared to the GHE group, there were fewer female patients (*p* = .045) and fewer non-Hispanic white patients (*p* = .04) in the CBT group. No other significant difference in patient characteristics was found between the two groups. The two groups were not significantly different in terms of caregiver characteristics except for hours of caregiving per week. The caregivers in the CBT group spent significantly more time providing care compared to the caregivers in the GHE group (*p* = .031).

**Table 2:** Baseline characteristics of the study population

	Patients		*p*-value	Family caregivers		*p*-value
	GHE group (*N* = 12)	CBT group (*N* = 14)		GHE group (*N* = 12)	CBT group (*N* = 14)	
Age (in years)	67.41 ± 17.14	66.54 ± 11.33	.88	54.46 ± 17.61	55.62 ± 13.54	.85
Female	9 (75.0)	5 (35.7)	.045	4 (33.3)	10 (71.4)	.052
Non-Hispanic white	7 (58.3)	2 (14.3)	.04	6 (50.0)	4 (28.6)	.42
Married or cohabitating	8 (66.7)	11 (78.6)	.67	6 (50.0)	12 (85.7)	.09
Education level			.07			.70
≤ High school	3 (25.0)	9 (64.3)		4 (33.3)	6 (42.9)	
Household income			.85			.51
< $20,000/year	3 (25.0)	3 (21.4)		3 (25.0)	2 (14.3)	
≥ $20,000/year	5 (41.7)	8 (57.1)		5 (41.7)	9 (64.3)	
Decline to state	4 (33.3)	3 (21.4)		4 (33.3)	3 (21.4)	
Employed	1 (8.3)	3 (21.4)	.60	3 (25.0)	7 (50.0)	.25
NYHA class			.40			
II	6 (50.0)	6 (42.9)				
III	6 (50.0)	6 (42.9)				
IV	0 (0.0)	2 (14.3)				
Charlson comorbidity index score	3.75 ± 2.01	3.57 ± 1.91	.94			
Spouse of patient				6 (50.0)	9 (64.3)	.46
Living with patient				11 (91.7)	12 (85.7)	1.00
Sharing caregiver role with others				5 (41.7)	10 (71.4)	.13
Duration of caregiving (in months)				57.55 ± 56.48	48.43 ± 68.79	.73
Hours of caregiving per week				2.08 ± 0.90	3.00 ± 1.11	.03
**Salivary cortisol**						
Cortisol awakening response^a^				0.119 ± 0.221	0.098 ± 0.282	.85
Area under the curve^b^				2096.3 ± 267.7	1978.6 ± 244.9	.31
**Psychological measures**^**c**^						
Caregiver burden (ZBI)				12.58 ± 7.35	13.43 ± 10.32	.82
Perceived stress (PSS-10)				15.83 ± 6.71	17.00 ± 7.93	.69
Stress symptoms (cSOSI)				35.83 ± 34.32	42.86 ± 40.02	.64
Depressive symptoms (PHQ-9)	10.36 ± 7.12	7.79 ± 4.90	.30	5.75 ± 6.38	7.50 ± 7.10	.52
Clinical depression (PHQ-9 score ≥ 10)	5 (41.67)	6 (42.9)	1.00	3 (25.0)	4 (28.6)	1.00
Anxiety (GAD-7)				4.92 ± 5.60	6.36 ± 6.05	.54

Note. Continuous variables are reported as mean ± SD. Categorical variables are reported as number (percentage) of patients or caregivers

*CBT* cognitive behavioral therapy, *cSOSI* Calgary Symptoms of Stress Inventory, *GHE* general health education, *GAD-7* Generalized Anxiety Disorder 7-item, *NYHA* New York Heart Association, *PHQ-9* Patient Health Questionnaire 9-items, *PSS-10* 10-item Perceived Stress Scale, *ZBI* Zarit Burden Interview

^a^The cortisol awakening responses was computed as the difference in log-transformed cortisol values from waking to 30 min post-awakening

^b^The area under the curve was computed using log-transformed cortisol values. The bedtime cortisol value was not available for 1 caregiver (GHE group) at baseline

^c^Higher scores indicate worse outcomes

### Adherence and attrition

The participation rate did not significantly differ between the groups. In the GHE group, 80% received at least 1 session and 73% received all 8 sessions. In the CBT group, 93% participated in at least 1 session and 73% completed all 8 sessions. The care recipient of one caregiver assigned to the CBT group deceased after the first session. Because the caregiver chose to continue the intervention, the remaining intervention sessions were provided. However, the intervention was modified to meet the caregiver’s needs and no follow-up data were collected from this caregiver. Additionally, the caregiver was referred to the primary care provider and joined a community grief support group.

On average, each GHE session lasted for 62.78 (SD 14.61) minutes, and each CBT session lasted for 61.16 (SD 14.95) minutes (*p* = .47). A total of 9 (60%) caregivers in the GHE group and 9 (60%) caregivers in the CBT group completed all follow-up assessments (*p* = 1.00).

### Outcomes

In linear mixed models, time-by-study group interactions were not significant for primary (i.e., CAR and AUC) or secondary outcomes (i.e., caregiver burden, perceived stress, stress symptoms, depression, and anxiety) (Table 3). However, the CBT group had significant improvements from baseline to post-intervention in perceived stress (*p* = .011), stress symptoms (*p* = .017), depression (*p* = .002), and anxiety (*p* = .006). Additionally, the CBT group showed significant improvements from baseline to 6 months in perceived stress (*p* = .034), stress symptoms (*p* = .043), depression (*p* = .046), and anxiety (*p* = .010). The GHE group showed a significant change only in anxiety from baseline to post-intervention (*p* = .030), and no other significant changes were found in the GHE group. The significance of the findings did not change when covariates (i.e., caregiver gender, caregiver marital status, and hours of caregiving per week) were added to each model.

**Table 3:** Longitudinal analyses of outcome measures by study group

	General health education group (*N* = 12)				Cognitive behavioral therapy group (*N* = 14)				*p*-value for interaction^c^
	Estimate^a^ (95% CI)			*p*-value^b^	Estimate^a^ (95% CI)			*p*-value^b^	
Cortisol awakening response^d^									.95
Baseline to post-intervention	0.02	(−0.13,	0.16)	.82	0.02	(−0.13,	0.17)	.76	
Area under the curve^e^									.12
Baseline to post-intervention	−137.03	(− 312.20,	38.15)	.12	53.44	(− 121.73,	228.61)	.53	
Caregiver burden									.97
Baseline to post-intervention	−2.16	(−5.53,	1.21)	.20	−1.71	(−5.22,	1.79)	.33	
Baseline to 6 mo	−2.41	(−7.18,	2.37)	.31	−1.65	(−6.46,	3.15)	.49	
Perceived stress									.30
Baseline to post-intervention	−1.08	(−4.19,	2.03)	.48	−4.29	(−7.52,	−1.07)	.011	
Baseline to 6 mo	−0.51	(−4.87,	3.86)	.82	−4.76	(−9.14,	− 0.38)	.034	
Stress symptoms									.097
Baseline to post-intervention	−1.15	(−9.52,	7.23)	.78	−10.86	(−19.61,	−2.11)	.017	
Baseline to 6 mo	6.28	(−5.98,	18.54)	.31	−12.90	(−25.36,	−0.45)	.043	
Depression									.26
Baseline to post-intervention	−1.05	(−3.15,	1.06)	.32	−3.54	(−5.73,	−1.35)	.002	
Baseline to 6 mo	−0.97	(−3.96,	2.03)	.52	−3.07	(−6.08,	−0.06)	.046	
Anxiety									.56
Baseline to post-intervention	−1.97	(−3.70,	−0.24)	.03	−2.58	(− 4.38,	−0.78)	.006	
Baseline to 6 mo	−1.46	(−3.93,	1.01)	.24	−3.28	(−5.77,	−0.78)	.01	

^a^Estimated changes from baseline to 2 months or 6 months derived from linear mixed models; negative values for psychological measures indicate improvement from baseline

^b^*p*-values are for tests of no change in outcome measures in each group

^c^*p*-values are for the time-by-study group interaction from the linear mixed models

^d^The cortisol awakening response was computed as the difference in log-transformed cortisol values from waking to 30 min post-awakening

^e^The area under the curve was computed using log-transformed cortisol values. The bedtime cortisol value was not available for 1 caregiver (GHE group) at baseline

Table 4 presents the outcome values at each follow-up assessment and mean change scores from baseline to post-intervention and 6 months for each outcome by study group as well as the effect size for the difference between groups in change for each outcome. The effect sizes (Cohen’s *d*) were very small for the between-group difference in change for caregiver burden and CAR (*d* = 0.04–0.10) and medium to larger for the between-group difference in change for perceived stress, stress symptoms, depression, and AUC (*d* = 0.53–1.06). For anxiety, the effect size was close to small for the between-group difference in change from baseline to post-intervention (*d* = 0.17) and close to medium for the between-group difference in change from baseline to 6-months (*d* = 0.75).

**Table 4:** Outcome scores at follow-up, mean change scores by study group, and effect sizes

	Post-intervention			6-month follow-up			Baseline to post-intervention			Baseline to 6-month follow-up		
	*N*	Mean	SD	*N*	Mean	SD	Mean change^a^	SD	Effect size (95% CI)^b^	Mean change^a^	SD	Effect size (95% CI)^b^
Cortisol awakening response^c^									−0.06			
GHE group	11	0.14	0.20				0.016	0.17	(−0.91,			
CBT group	10	0.14	0.14				0.003	0.27	0.80)			
Area under the curve^d^									0.78			
GHE group	10	1993.19	362.53				− 161.93	343.33	(−0.16,			
CBT group	10	2050.94	238.58				39.57	131.53	1.65)			
Caregiver burden									0.10			−0.04
GHE group	11	11.36	6.77	9	10.56	7.16	−2.36	5.37	(−0.76,	−1.89	2.67	(−0.96,
CBT group	10	12.10	9.05	9	11.56	10.56	−1.80	6.32	0.95)	−2.11	7.82	0.89)
Perceived stress									−0.53			−0.81
GHE group	11	15.45	6.38	9	15.00	8.75	−1.27	6.60	(−1.38,	0.11	6.66	(−1.73,
CBT group	10	12.00	8.03	9	12.56	8.63	−4.10	3.31	0.36)	−4.44	4.36	0.19)
Stress symptoms									−0.67			−1.06
GHE group	11	36.45	35.68	9	32.44	34.82	−1.27	13.88	(−1.52,	3.78	18.43	(−1.99,
CBT group	10	29.14	26.71	9	28.44	30.33	−10.66	14.01	0.23)	−13.67	14.21	−0.03)
Depression									−0.53			−1.04
GHE group	11	4.91	3.96	9	4.11	4.81	−1.09	4.74	(−1.38,	0.33	2.06	(−1.97,
CBT group	10	3.30	4.42	9	4.00	4.30	−3.40	3.86	0.36)	−3.33	4.56	−0.01)
Anxiety									−0.17			−0.75
GHE group	11	3.09	4.04	9	2.67	3.00	−2.00	4.10	(−1.02,	−0.89	3.89	(−1.66,
CBT group	10	3.90	4.20	9	3.44	3.68	−2.60	2.88	0.70)	−3.56	3.24	0.24)

*CBT* cognitive behavioral therapy, *GHE* general health education

^a^Mean change scores were computed by subtracting the baseline score from the follow-up score using pairwise deletion: i.e., post-intervention score – baseline score; 6-month score – baseline score

^b^Effect sizes (Cohen’s *d*) were computed by subtracting the mean change scores in the GHE group from the mean change scores in the CBT group and dividing the result by the pooled SD: i.e., (mean change in the CBT group – mean change in the GHE group)/pooled SD

^c^The cortisol awakening response was computed as the difference in log-transformed cortisol values from waking to 30 min post-awakening

^d^The area under the curve was computed using log-transformed cortisol values. The bedtime cortisol value was not available for 1 caregiver (GHE group) at baseline

## Discussion

This is one of the few studies testing the effects of an intervention designed for family caregivers of patients with HF. Despite the known detrimental effects of caregiving on the physical and mental health of family caregivers, there is limited evidence for effective interventions for HF caregivers [2]. In this study, we examined the preliminary effects of CBT on outcomes of HF caregivers. Overall, there was no significant difference in the rate of change in all outcome measures between the two groups, which may be attributable to the small sample size. In other words, CBT was not superior to GHE for primary (physiological) outcomes (i.e., CAR and AUC) or secondary (psychological) outcomes (i.e., caregiver burden, perceived stress, stress-related symptoms, depression, and anxiety).

To our knowledge, this is one of the first studies that examined the effect of an intervention using a biomarker in HF caregivers. Despite the strong need for objective measures of caregiver stress, biomarkers have been used in only a limited number of caregiver studies and used even less frequently in caregiver intervention studies. In our study, there was no significant intervention effect on cortisol indices. Previous caregiver intervention studies have shown mixed findings regarding intervention effects on cortisol. In a systematic review of interventions for dementia caregivers, Allen et al. [10] identified 18 studies in which intervention effects were examined using a biomarker. Salivary cortisol was measured in 5 of those studies, and a significant intervention effect on cortisol was observed in only 2 studies. In another systematic review of studies that measured pro-inflammatory biomarkers in caregivers of older individuals, 24 articles were identified [13]. Most of the identified studies were cross-sectional, and only 3 were randomized controlled trials testing the effect of caregiver interventions. Salivary cortisol was assessed in 2 of the studies, and the findings were inconsistent. Therefore, more research is needed to draw conclusions about the effects of caregiver interventions on salivary cortisol.

In addition, although not statistically significant, the total cortisol output (i.e., AUC) was increased in the CBT group and was decreased in the GHE group in our study. These findings are contrary to our hypothesis and warrant further research to explore the impact of chronic stress on the HPA axis activity in the context of HF caregiving. Increased cortisol levels have been reported in caregivers of individuals with dementia [10]. However, recent studies have shown either no difference in the cortisol levels and CARs between caregivers and non-caregivers [14] or hypocortisolism in mothers of children with autism spectrum disorder [37]. In these recent studies, hypocortisolism was viewed as a result of a compensatory mechanism in response to chronic stress [37, 38]. The nature of caregiving may be different depending on the care recipient’s type of illness, stage in the disease trajectory, and caregiver’s relation to the care recipient. Therefore, the long-term impact of caregiving on the HPA axis activity needs further exploration in the context of HF.

In our study, the CBT group showed significant immediate improvements in all psychological outcomes except for caregiver burden (i.e., perceived stress, stress symptoms, depression, and anxiety), whereas the GHE group had no significant within-group change in the outcomes except for anxiety. The significant improvements in psychological outcomes observed in the CBT group also lasted for 6 months. In addition, medium to large effect sizes were observed for between-group differences in change for psychological outcomes except for caregiver burden. The effect size was larger for the change from baseline to 6-months compared to the change from baseline to post-intervention, indicating that the intervention effects on psychological outcomes increased after completion of the intervention. These findings suggest that CBT has potential for reducing psychological distress in HF caregivers and need to be confirmed in larger trials with a longer follow-up.

Accumulating evidence has indicated that CBT can effectively reduce psychological distress in caregivers of individuals with chronic illnesses, including dementia and cancer [4, 5, 39, 40]. Similar to our findings, in a meta-analysis of 11 randomized controlled trials of CBT for dementia caregivers, Vernooij-Dassen et al. [5] reported that CBT was effective in reducing caregiver depression, anxiety, and perceived stress, not in decreasing caregiver burden. In addition, reviewing meta-analyses of psychological interventions (including CBT) that aimed to reduce caregiver burden and distress, Adelman et al. [4] concluded that the interventions effectively improved caregiver depression, anxiety, and self-efficacy even when the intervention had no significant effect on caregiver burden itself. Based on their consistent effects on psychological symptoms that caregivers frequently experience, psychological interventions are generally recommended for caregivers of individuals with chronic illnesses [4, 5, 39, 40]. However, according to the recent scientific statement from the American Heart Association [2], evidence is still inadequate to make any recommendations for caregivers of individuals with HF. More high-quality studies are needed to establish the effectiveness of psychological interventions for HF caregivers.

Previous studies have shown inconsistent findings regarding the effects of psychological interventions on caregiver burden. In a recent meta-analysis of interventions for dementia caregivers, Kishita et al. [41] reported that psychoeducation skill-building interventions had no significant effect on caregiver depression and a small effect on caregiver burden, whereas CBT-based interventions had a moderate effect on caregiver depression and no significant effect on caregiver burden. Reviewing 30 randomized controlled trials of interventions designed to reduce burden among dementia caregivers, Williams et al. [42] concluded that multi-component interventions were more effective in reducing caregiver burden than education/skill, support/counseling, and physical activity interventions. CBT techniques or teaching of relevant coping strategies were part of the multi-component interventions that showed significant effects on caregiver burden. The findings from these meta-analyses suggest that CBT-based interventions are effective in reducing caregiver depression, but additional strategies, such as psychoeducation and social support, are needed to decrease caregiver burden.

In this study, both CBT and GHE were delivered via home visits to minimize participant burden. Home visits and face-to-face interventions generally require substantial clinician time and effort, which leads to increased costs. Although the evidence shows the efficacy of CBT delivered via computer or mobile device, concerns still remain, such as low user engagement rates, poor access to technology in disadvantaged and older populations, and lack of evidence on the effects beyond the intervention period [43, 44]. Future studies are needed to determine whether other interventions that are simple or less labor-intensive (e.g., computer-based CBT, mobile-application based CBT) have comparable effects.

There are a few limitations of this study that should be considered when interpreting the findings. First, our study was designed as a pilot study, and therefore, the findings should be interpreted as preliminary. Despite efforts made to meet the enrollment target of the study, recruitment fell short of the target. Some of the non-significant findings, including the non-significant time-by-study group interactions, may be due to the small sample size. Further large trials are needed to confirm the effects of CBT on outcomes of HF caregivers. Second, because the study sample was recruited from 2 university-affiliated hospitals located in an urban area, the generalizability of our findings is limited to similar settings. Third, the control group in our study received 8 sessions of GHE in order to control for the benefits of attention. However, the GHE may have helped caregivers in the control group engage in health promoting behaviors and may have led to underestimation of effect sizes. This possibility can be explored in future research comparing CBT combined with GHE against GHE only. Fourth, we relied on caregivers to collect saliva samples and record the timing of sample collection. Therefore, we cannot rule out the possibility of non-compliance with the procedures and off-time assessments, which may have affected CAR estimates. Although caregivers in our study received detailed instructions on sample collection and reminders the night before each sample collection, additional strategies, such as using electronic monitoring devices, could have ensured better adherence to the sampling protocol [45].

## Conclusions

Despite the known negative impact of caregiving on the physical and mental health of family caregivers, there have been only a limited number of studies that have examined the effect of interventions for family caregivers of individuals with HF. Although no significant between-group differences in change were observed in our study, the CBT group had significant improvements in some psychological outcomes with no improvement in the control group. Our findings suggest that the CBT can potentially reduce psychological distress in HF caregivers. Because our findings are preliminary, larger randomized controlled trials are necessary to test the efficacy of CBT and examine its effect on the HPA axis activity (i.e., salivary cortisol) in the context of HF. Studies are also needed to further explore the impact of HF caregiving on the HPA axis activity.

## Data Availability

The data presented in this paper are available from the corresponding author upon reasonable request.

## Abbreviations

AUC: The area under the curve
CAR: Cortisol awakening response
CBT: Cognitive behavioral therapy
cSOSI: Calgary Symptoms of Stress Inventory
GAD-7: Generalized Anxiety Disorder 7-item
GHE: General health education
HF: Heart failure
HPA axis: Hypothalamic-pituitary-adrenal axis
NYHA class: New York Heart Association class
PHQ-9: Patient Health Questionnaire 9-item
PSS-10: Perceived Stress Scale 10-item
ZBI: Zarit Burden Interview
